# Supplementary figures and images for: Evidence of pandemic fatigue associated with stricter tiered COVID-19 restrictions
Source: PLOS Digit Health. 2022 May 26;1(5):e0000035. doi: 10.1371/journal.pdig.0000035 (PMC9931343; doi:10.1371/journal.pdig.0000035)

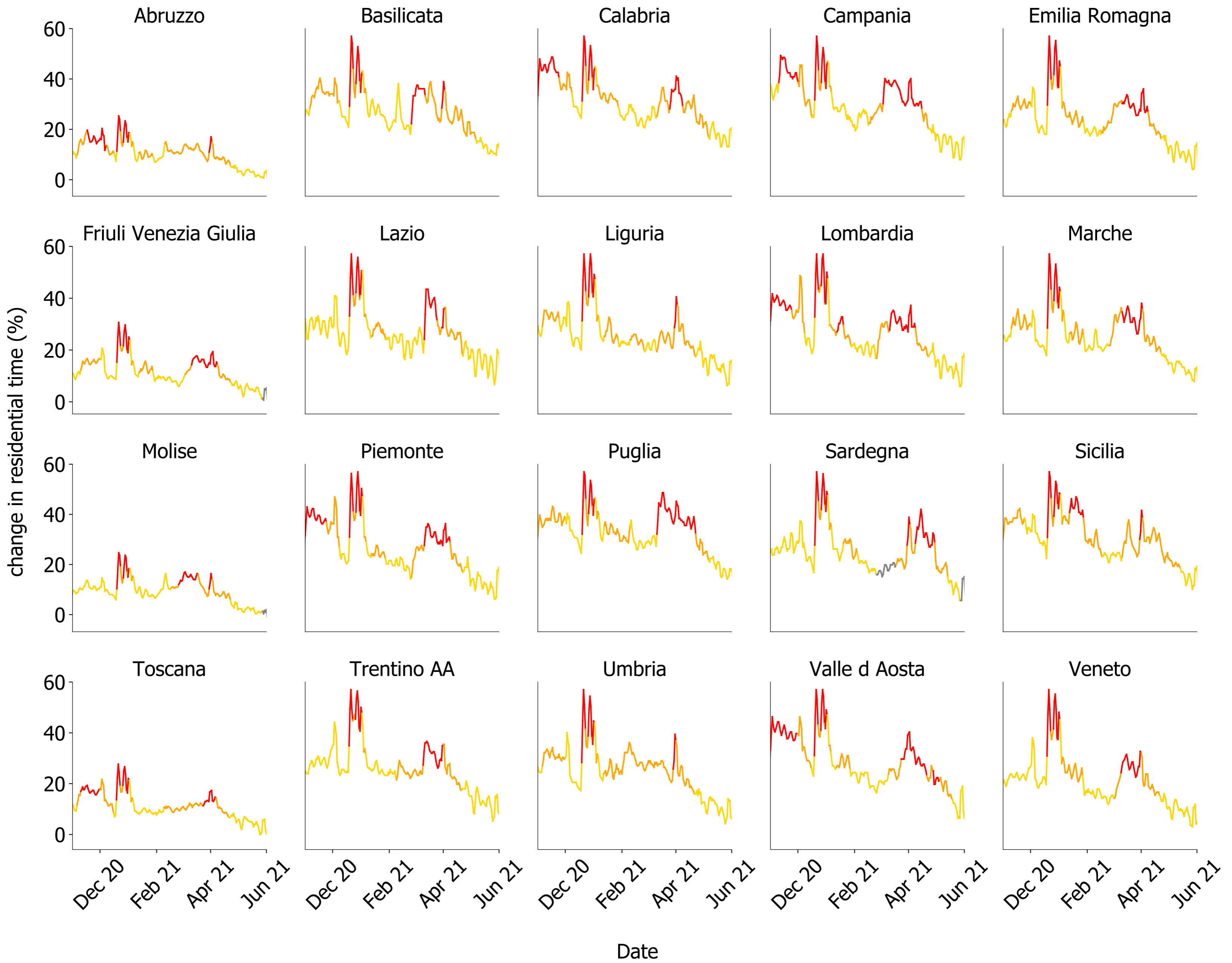

Supplement: S1 Fig — Daily relative change in the residential time—as measured by Google—with respect to baseline, in the Italian regions ordered alphabetically from top to bottom. Color coding indicates the tier that was in place each day. Gray indicates the absence of restrictions. (PDF) [file pdig.0000035.s001.pdf]
