# Supplementary material for: Evidence of pandemic fatigue associated with stricter tiered COVID-19 restrictions
Source: PLOS Digit Health. 2022 May 26;1(5):e0000035. doi: 10.1371/journal.pdig.0000035 (PMC9931343; doi:10.1371/journal.pdig.0000035)
Supplement: S1 Table — (PDF) [file pdig.0000035.s002.pdf]

	Dependent variable:			
	Relative change in mobility (%)			
	(1)	(2)	(3)	(4)
Basilicata	−0.161	−0.459	−0.474	−0.103
	(0.999)	(1.119)	(1.120)	(1.000)
Calabria	2.127**	2.212**	2.105*	2.174**
	(0.999)	(1.120)	(1.121)	(1.001)
Campania	0.214	−0.076	−0.554	−0.097
	(1.008)	(1.130)	(1.139)	(1.017)
Emilia Romagna	2.577***	2.756**	2.539**	2.486**
	(0.999)	(1.120)	(1.121)	(1.001)
Friuli Venezia Giulia	1.120	1.161	0.909	1.037
	(1.002)	(1.123)	(1.125)	(1.004)
Lazio	−1.782*	−2.221**	−2.345**	−1.704*
	(1.007)	(1.127)	(1.133)	(1.011)
Liguria	2.348**	2.688**	2.503**	2.321**
	(1.001)	(1.124)	(1.124)	(1.004)
Lombardia	0.013	0.287	0.066	0.007
	(1.004)	(1.125)	(1.129)	(1.008)
Marche	−0.177	0.019	−0.163	−0.201
	(1.000)	(1.122)	(1.123)	(1.002)
Molise	−1.938*	−2.368**	−2.528**	−1.888*
	(1.006)	(1.126)	(1.132)	(1.011)
Piemonte	0.338	0.552	0.296	0.274
	(1.003)	(1.124)	(1.127)	(1.006)
Puglia	0.968	0.923	0.505	0.592
	(1.000)	(1.120)	(1.127)	(1.006)
Sicilia	1.972**	2.247**	2.238**	1.998**
	(0.999)	(1.119)	(1.118)	(0.998)
Toscana	0.329	0.193	0.177	0.382
	(1.000)	(1.120)	(1.119)	(0.999)
Umbria	0.270	−0.769	−0.393	0.196
	(1.001)	(1.132)	(1.146)	(1.023)
Valle_d_Aosta	4.925***	5.171***	4.865***	4.838***
	(1.005)	(1.126)	(1.131)	(1.010)
Veneto	2.508**	2.123*	1.957*	2.512**
	(1.004)	(1.124)	(1.128)	(1.007)
Note:				*p<0.1; **p<0.05; ***p<0.01
