# Supplementary material for: Evidence of pandemic fatigue associated with stricter tiered COVID-19 restrictions
Source: PLOS Digit Health. 2022 May 26;1(5):e0000035. doi: 10.1371/journal.pdig.0000035 (PMC9931343; doi:10.1371/journal.pdig.0000035)
Supplement: S2 Table — (PDF) [file pdig.0000035.s003.pdf]

	Dependent variable:			
	Change in residential time (%)			
	(1)	(2)	(3)	(4)
Basilicata	0.021	0.154	0.214	0.059
	(0.254)	(0.353)	(0.352)	(0.254)
Calabria	−0.595**	−0.640*	−0.534	−0.557**
	(0.254)	(0.353)	(0.353)	(0.254)
Campania	−0.169	−0.042	0.185	−0.007
	(0.257)	(0.356)	(0.358)	(0.258)
Emilia Romagna	0.595**	0.497	0.603*	0.638**
	(0.254)	(0.353)	(0.353)	(0.254)
Friuli Venezia Giulia	1.494***	1.449***	1.589***	1.554***
	(0.255)	(0.354)	(0.354)	(0.255)
Lazio	1.444***	1.613***	1.763***	1.513***
	(0.256)	(0.355)	(0.356)	(0.257)
Liguria	0.100	−0.091	0.005	0.109
	(0.255)	(0.354)	(0.354)	(0.255)
Lombardia	1.817***	1.681***	1.837***	1.872***
	(0.255)	(0.355)	(0.355)	(0.256)
Marche	0.483*	0.366	0.474	0.510**
	(0.255)	(0.354)	(0.353)	(0.255)
Molise	0.138	0.308	0.475	0.222
	(0.256)	(0.355)	(0.356)	(0.257)
Piemonte	0.865***	0.753**	0.906**	0.927***
	(0.255)	(0.354)	(0.355)	(0.256)
Puglia	−0.251	−0.231	−0.101	−0.140
	(0.255)	(0.353)	(0.355)	(0.256)
Sicilia	−1.324***	−1.455***	−1.450***	−1.343***
	(0.254)	(0.353)	(0.352)	(0.254)
Toscana	0.689***	0.746**	0.792**	0.709***
	(0.255)	(0.353)	(0.352)	(0.254)
Trentino Alto Adige	1.383***	1.565***	1.686***	1.454***
	(0.255)	(0.355)	(0.354)	(0.255)
Umbria	0.382	0.894**	0.622*	0.338
	(0.255)	(0.357)	(0.360)	(0.260)
Valle d'Aosta	0.290	0.176	0.363	0.378
	(0.256)	(0.355)	(0.356)	(0.257)
Veneto	0.613**	0.765**	0.909**	0.690***
	(0.256)	(0.355)	(0.355)	(0.256)

Note: *p<0.1; **p<0.05; ***p<0.01
