# Supplementary material for: Evidence of pandemic fatigue associated with stricter tiered COVID-19 restrictions
Source: PLOS Digit Health. 2022 May 26;1(5):e0000035. doi: 10.1371/journal.pdig.0000035 (PMC9931343; doi:10.1371/journal.pdig.0000035)
Supplement: S3 Table — (PDF) [file pdig.0000035.s004.pdf]

	Dependent variable:			
	Change in movement (%)			
	(1)	(2)	(3)	(4)
Global time trend				
$\gamma_{1,0}$	0.082***	0.098***	0.085***	0.084***
	(0.003)	(0.003)	(0.003)	(0.003)
Local time trend				
$\gamma_{2,0}$	0.155***	0.232***	0.170***	0.166***
	(0.045)	(0.044)	(0.045)	(0.045)
$\gamma_{2,1}$ (orange)	−0.149***	−0.233***	−0.167***	−0.161***
	(0.052)	(0.050)	(0.052)	(0.052)
$\gamma_{2,1}$ (yellow)	−0.165***	−0.245***	−0.180***	−0.178***
	(0.051)	(0.049)	(0.051)	(0.051)
Epidemiological covariate				
β_3 (daily reported cases)		0.004***		
		(0.0002)		
β_3 (daily hospitalizations)			0.013***	
			(0.003)	
β_3 (daily ICU)				0.042***
				(0.014)
Intercept				
$\gamma_{0,0}$	−41.047***	−46.478***	−42.525***	−41.891***
	(0.993)	(1.005)	(1.063)	(1.030)
$\gamma_{0,1}$ (orange)	11.343***	13.910***	12.009***	11.815***
	(0.770)	(0.754)	(0.788)	(0.785)
$\gamma_{0,1}$ (yellow)	19.990***	23.177***	20.879***	20.609***
	(0.794)	(0.785)	(0.826)	(0.820)
Observations	3,222	3,222	3,222	3,222
R^2	0.458	0.502	0.461	0.460
Adjusted R^2	0.454	0.498	0.457	0.456
AIC	23,631	23,361	23,619	23,624
Note:				*p<0.1; **p<0.05; ***p<0.01
