# Supplementary material for: Evidence of pandemic fatigue associated with stricter tiered COVID-19 restrictions
Source: PLOS Digit Health. 2022 May 26;1(5):e0000035. doi: 10.1371/journal.pdig.0000035 (PMC9931343; doi:10.1371/journal.pdig.0000035)
Supplement: S4 Table — (PDF) [file pdig.0000035.s005.pdf]

	Dependent variable:			
	Change in residential time (%)			
	(1)	(2)	(3)	(4)
**Global time trend**				
$\gamma_{1,0}$	-0.039^{***}	-0.039^{***}	-0.038^{***}	-0.039^{***}
	(0.001)	(0.001)	(0.001)	(0.001)
**Local time trend**				
$\gamma_{2,0}$	-0.045^{***}	-0.043^{***}	-0.040^{***}	-0.043^{***}
	(0.011)	(0.011)	(0.011)	(0.011)
$\gamma_{2,1}$ (orange)	0.051^{***}	0.049^{***}	0.046^{***}	0.049^{***}
	(0.013)	(0.013)	(0.013)	(0.013)
$\gamma_{2,1}$ (yellow)	0.028^{**}	0.026^{**}	0.023^{*}	0.026^{**}
	(0.013)	(0.013)	(0.013)	(0.013)
**Epidemiological covariate**				
β_3 (daily reported cases)		0.0001^{*}		
		(0.0001)		
β_3 (daily hospitalizations)			0.004^{***}	
			(0.001)	
β_3 (daily ICU)				0.007^{**}
				(0.003)
**Intercept**				
$\gamma_{0,0}$	19.225^{***}	19.083^{***}	18.790^{***}	19.081^{***}
	(0.250)	(0.264)	(0.267)	(0.259)
$\gamma_{0,1}$ (orange)	-4.628^{***}	-4.561^{***}	-4.434^{***}	-4.548^{***}
	(0.193)	(0.196)	(0.197)	(0.196)
$\gamma_{0,1}$ (yellow)	-7.520^{***}	-7.436^{***}	-7.260^{***}	-7.414^{***}
	(0.198)	(0.204)	(0.205)	(0.204)
Observations	3,401	3,401	3,401	3,401
R^2	0.709	0.710	0.711	0.710
Adjusted R^2	0.707	0.707	0.709	0.708
AIC	15,626	15,625	15,607	15,623

Note: $^{*}p<0.1$; $^{**}p<0.05$; $^{***}p<0.01$
