# Supplementary material for: Evidence of pandemic fatigue associated with stricter tiered COVID-19 restrictions
Source: PLOS Digit Health. 2022 May 26;1(5):e0000035. doi: 10.1371/journal.pdig.0000035 (PMC9931343; doi:10.1371/journal.pdig.0000035)
Supplement: Attachment — Submitted filename: Response_reviewers.pdf [file pdig.0000035.s007.pdf]

Reviewer #1

I like this paper. It asks a critically important question: does compliance with public health measures wane with time and with the stringency of the measures. If it does, then public health measures need to take account of waning. If it doesn't, then officials who sometimes hold fire for fear of waning effects could be more confident in setting restrictions where appropriate.

We thank the referee for his very positive assessment of our work. In the following, we reply to the two main concerns he raised, into two separate parts.

1. **Spontaneous behavior**

Covid restrictions are themselves endogenous to actual Covid levels. So when things start looking dangerous, the restrictions come in. When they start looking safe again, restrictions ease. But restrictions are a discrete value and the amount of Covid in the community is continuous. People will have *started* taking their own protective measures ahead of the restrictions, and will have *started* taking fewer precautions before the restrictions are eased. On the downside of that curve, are we seeing waning compliance with the measure because they're sick of the measures? Or are we seeing waning compliance because people are judging the world to look less risky and that, in their view, the measures are now less warranted? It will be a mix of the two. Disentangling it requires having some measure of the perceived riskiness of the environment.

I think the statistical model needs to incorporate actual Covid prevalence in each place over time as a control - unless actual Covid levels in each region were the same at each point in time and only the restrictions varied. If that's the case, then regional variation in restrictions could identify. But I rather strongly suspect that regional restrictiveness will depend on regional Covid levels.

If measures like number of positive tests or proportion of positive tests aren't available at the finer-grained local level, the local hospital burden could be a pretty good proxy. If the hospitals are overflowing, I'm staying home regardless of restrictions. Once the hospitals are running normally again, I'll be less inclined to comply with restrictions - nothing to do with frustration about restrictions, just that "Don't swim in the river" signs are superfluous when the river is a raging torrent, but are likely to be ignored on a hot day by a calm and beautiful swimming spot. Changing levels of compliance with the "Do Not Swim" sign in that case have nothing to do with waning support for the sign's restrictions as people become frustrated with it and everything to do with people's own assessment of whether the sign's warning is reasonable in the circumstances.

We understand the concern and we followed the suggestion by the referee. To control for the perceived riskiness of the environment, we have added to the full model an epidemiological covariate as a control variable.

We consider all the epidemiological covariates that are available for the Italian regions: the daily reported number of positive cases by region, the daily number of new hospitalizations, and daily ICU admissions. The results are robust to any of these additional covariates. Indeed our statistical analysis still shows that changes in adherence were faster during periods characterized by the strictest level of restrictions (the red tier) and the magnitude of the two

effects - the decrease in adherence over time and specifically during the red tier - was of a similar order.

We added a paragraph about these new results in the main manuscript and two tables (one for each mobility indicator) with the 3 new models in the Supplementary Information.

2. **Confounding factors and choice of mobility metrics**

The second concern probably cannot easily be addressed but should be listed as a potential limitation of the work. There was a thoroughly depressing paper published in the NBER working paper series in December on the use of mobility data in exactly this kind of work. See Weill, J, M Stigler, O Deschenes and M Springborn. 2021. "Researchers' Degrees-of-Flexibility and the credibility of Difference-in-Differences Estimates: Evidence from the Pandemic Policy Evaluations", NBER Working Paper 29550. https://www.nber.org/papers/w29550 . They show that the American literature on the effects of lockdowns on mobility is, in short, a mess. There are tons of ways of measuring mobility, and they don't correlate well with each other. They point to this as introducing huge researcher degrees of freedom: basically, motivated researchers can pick the result they want by choosing the mobility measure that gives them that result. I have absolutely no reason to expect that this has happened in
this paper. But it points to a danger in this work: none of the mobility measures are themselves very good if the choice of measure winds up substantially influencing whether restrictions work or not. To my mind, that argues for using a broad set of mobility measures, running a principal components analysis through it, seeing whether there's some underlying measure on which they all load, and use that factor as the measure of mobility. That would be a ridiculous amount of work here, and I do not know whether those additional measures are even available for Italy. But readers should be given a pretty strong warning that, in the American literature, choice of mobility measure can prove strongly influential on the results and that the work should be replicated using other mobility measures to check consistency. I also note that the NBER work uses a much broader array of controls looking at effects on mobility: precipitation, snowfall, mean temperatures. The paper is really worth going through closely.

We thank the reviewer for pointing us to the report by Weill, J, M Stigler, O Deschenes, and M Springborn. In this paper, the authors say that event-study estimates are highly sensitive to choices of mobility outcome, and we indeed agree that different mobility outcomes capture different facets of mobility.

That is why we looked at two indicators of mobility. The two indicators capture two different and complementary aspects of mobility: while Facebook measures the change in the number of movements, Google estimates the change in time spent at home, thus providing an indicator of stillness.

We found evidence for pandemic fatigue in both indicators, however we argue that not finding this evidence for one of the mobility indicators would not have compromised the robustness of our results as the pandemic fatigue might reflect only in some aspects of mobility. For instance, people might decide to go outside more but still go to only a few places.

In the future, it could be interesting to extend our study using other mobility outcomes that can be derived from mobile phone data, such as spatial proximity or the radius gyration.

We expanded the discussion to include a paragraph with the above considerations.

Reviewer #2

I appreciate the authors for the analysis performed and the manuscript is well written. The statistical methodology used in this study are appropriate.
The results are found to be satisfactory.
I would recommend to check for the grammatical errors and rephrasing few sentences.

We thank the referee for the positive comment and for considering our methods appropriate. We revised the manuscript and carefully checked for grammatical errors.

Reviewer #3

The study "Evidence of pandemic fatigue associated with stricter tiered COVID-19 restrictions" uses mobility data to parameterize adherence to tiered interventions over time in Italy accounting for social distancing measures that were in place in every region each day. Results of the study are of interest to public health and can be integrated into mathematical models to evaluate optimal intervention policies for future epidemics. Methods are sound, results adequately described and limitations acknowledged.
Tables and figures should be self-explanatory. Please explain coefficients' labels in the first column of tables 1 and 2.

We thank the referee for the very positive assessment of our manuscript.
We revised tables 1 and 2 by grouping the coefficients and adding short titles that explain their meaning.
